# Ochratoxin A and Its Role in Cancer Development: A Comprehensive Review

**DOI:** 10.3390/cancers16203473

**Published:** 2024-10-14

**Authors:** Magdalena Więckowska, Natalia Cichon, Rafał Szelenberger, Leslaw Gorniak, Michal Bijak

**Affiliations:** Biohazard Prevention Centre, Faculty of Biology and Environmental Protection, University of Lodz, Pomorska 141/143, 90-236 Lodz, Poland; magdalena.wieckowska@biol.uni.lodz.pl (M.W.); rafal.szelenberger@biol.uni.lodz.pl (R.S.); leslaw.gorniak@biol.uni.lodz.pl (L.G.); michal.bijak@biol.uni.lodz.pl (M.B.)

**Keywords:** ochratoxin A, cancer, nephrotoxicity, renal cell carcinoma, gastrointestinal cancer, hepatocellular carcinoma, rare neoplasma, immunosuppression, DNA damage, oxidative stress

## Abstract

**Simple Summary:**

Ochratoxin A (OTA) present in food products poses a serious threat to health due to its wide spectrum of effects, including genotoxicity, nephrotoxicity, neurotoxicity, embryotoxicity, teratogenicity, and immunosuppression. Moreover, it is associated with carcinogenesis, as a result of which it has been classified as a potential human carcinogen. In this review, literature reports on the correlations between carcinogenesis and OTA, with particular emphasis on certain organs such as the kidney or digestive system organs and the cancers associated with them, are gathered. Furthermore, we describe the potential mechanisms underlying OTA-induced carcinogenesis and discuss existing limitations.

**Abstract:**

**Background:** Ochratoxin A (OTA) is widely recognized for its broad spectrum of toxic effects and is classified as a potential human carcinogen, placed in group 2B by the International Agency for Research on Cancer (IARC). Its presence in food and beverages poses a significant health hazard. Extensive research has documented the efficient absorption and distribution of OTA throughout the body via the bloodstream and tissues, underscoring the associated health risk. Additionally, ongoing studies aim to clarify the link between OTA exposure and carcinogenesis. The obtained results indicate a strong correlation between OTA and renal cell carcinoma (RCC), with potential associations with other malignancies, including hepatocellular carcinoma (HCC), gallbladder cancer (GBC), and squamous cell carcinoma (SCC). OTA is implicated in oxidative stress, lipid peroxidation, apoptosis, DNA damage, adduct formation, miRNA deregulation, and distributions in the cell cycle, all of which may contribute to carcinogenesis. **Conclusions:** Despite significant research efforts, the topic remains inexhaustible and requires further investigation. The obtained results do not yield definitive conclusions, potentially due to species-specific differences in the animal models used and challenges in extrapolating these results to humans. In our review, we delve deeper into the potential mechanisms underlying OTA-induced carcinogenesis and discuss existing limitations, providing directions for future research.

## 1. Introduction

Ochratoxin A (OTA), produced by Aspergillus and Penicillium fungi, is known as the most toxic among ochratoxins. It can be synthesized in different climates depending on fungi species [1] and occur on cultivated cereals [2,3,4]. Many studies report its presence in many food products and beverages such as rice, maize, dry fruits, spices, herbs, nuts, beer, wine, and animal-derived products like milk, cheese, meat, and eggs [5,6,7,8,9,10,11,12,13,14,15,16,17]. Thus, humans are mainly exposed to OTA through dietary consumption [18].

The structure of OTA includes a phenylalanine connected via an amide bond to a dihydroisocoumarin ring [19], in which a chlorine atom is present and imparts its toxic properties [20]. The phenylalanine moiety’s carboxyl group and the isocoumarin part’s phenolic hydroxyl group have pKa values of 4.2–4.4 and 7.0–7.3, respectively, which are crucial for the adsorption of OTA. The monoanionic form (OTA^−^) and the dianionic form (OTA^2−^) occur in the duodenal chyme under physiological pH conditions, while the fully protonated toxin primarily occurs in acidic solutions such as the upper gastrointestinal tract [1].

Upon ingestion, OTA is absorbed through the gastrointestinal tract into the systemic circulation, where it binds to serum proteins, mainly albumin [1]. Schwerdt et al., in a study conducted on rats, reported that OTA has the capacity to bind to a wide range of proteins with varying degrees of specificity, as proteins with molecular masses between 55–60 kDa, 40–45 kDa, and 25–30 kDa demonstrated OTA binding across all samples. Furthermore, within the organellar compartment of an epithelial cell line from the proximal tubule of an adult female opossum (OK cell line), a 62 kDa protein was preferentially detected by OTA conjugated to horseradish peroxidase (OTA-HRP) [21]. Another way of distributing OTA in the body is tissue distribution [1,19], which is species-dependent and is also influenced by various factors, including the dose of the toxin, the route of administration, dietary composition, and the overall health status of the body [19]. The toxin is efficiently transported between tissues due to enterohepatic recirculation, which involves the reabsorption of OTA from the intestines back into the bloodstream. Additionally, OTA undergoes reabsorption in both the proximal and distal tubular regions of the kidneys. Consequently, OTA accumulates primarily in the bloodstream and organs such as the kidneys and liver, which are also the principal sites of its biotransformation [1], but can also be found in skeletal muscle, adipose tissue, and the brain [19].

The metabolism of OTA varies among species and results in several metabolites [22]. OTA undergoes biotransformation through phase I and II enzymatic processes [19]. OTA biotransformation occurs via at least two distinct mechanisms: enzymatic hydrolysis and cytochrome P450 (CYP450) induction [22]. While the majority of OTA metabolites are less toxic or non-toxic [19], the lactone-opened product, OP-OTA, exhibits greater toxicity than OTA itself [22]. The final step of biotransformation is excretion through urine, feces, and milk [1,19].

In animals, OTA is passively absorbed primarily in its non-ionized and monoanionic forms from the stomach and jejunum. The extent of absorption varies depending on the species, i.e.: rabbits, rats, and pigs have absorption rates of 40%, 56%, and approximately 60%, respectively. In comparison, absorption in humans reaches approximately 93% [1,19]. The peak concentrations of OTA in the blood were observed between 24 and 48 h post-administration, reaching approximately 4.6 µmol/L in males and 6.0 µmol/L in females. The elimination of OTA from the blood followed first-order kinetics, with a calculated half-life of approximately 230 h. In the liver of rats, the concentration of OTA remained below 12 pmol/g of tissue, regardless of sex, with the highest levels observed 24 h after administration. In the kidneys, the measured concentration was significantly elevated, reaching 480 pmol/g of tissue 24 h post-administration. Interestingly, OTA levels were consistently higher in male subjects compared to females [23]. Recently, Zhu et al. performed a study in which a single dose of OTA (500 µg/kg) was administered orally on lactating sows. Furthermore, the biological samples including plasma, stool, urine, and milk samples were collected at different time intervals. The results of the study showed that peak OTA plasma concentrations (920.25 ± 88.46 μg/L) occurred nine hours post-administration, with an elimination half-life of 78.47 ± 7.68 h. OTA was excreted in feces and urine over the 120-h period, representing 18.70 ± 0.04% of the total administered dose. Furthermore, OTA residues were detected in the milk, with the milk-to-plasma ratio increasing from 0.06 to 0.46 within the first 24 h following OTA ingestion [24]. In a similar study, donkeys were administered a single oral dose of 2500 μg/kg body weight of OTA, and plasma samples were collected at various time intervals. The results indicated that the peak plasma concentration of OTA, averaging 10.34 µg/mL, was observed 12 h post-administration. The total excretion of OTA through urine and feces accounted for approximately 10% of the administered dose over 120 h [25].

Due to the extensive toxic effects of OTA on various organs, ongoing research aims to elucidate its precise mode of action, as current findings remain inconclusive. Additionally, the ambiguity surrounding OTA’s carcinogenic potential in humans underscores the necessity for further investigation, as reflected by recent publications on this subject. This review article seeks to address these issues by compiling and summarizing research findings, with a particular focus on mechanisms of carcinogenicity. The goal is to advance understanding and contribute to the continued exploration of this critical topic.

## 2. Procarcinogenic Action of Ochratoxin A

**Figure 1:**
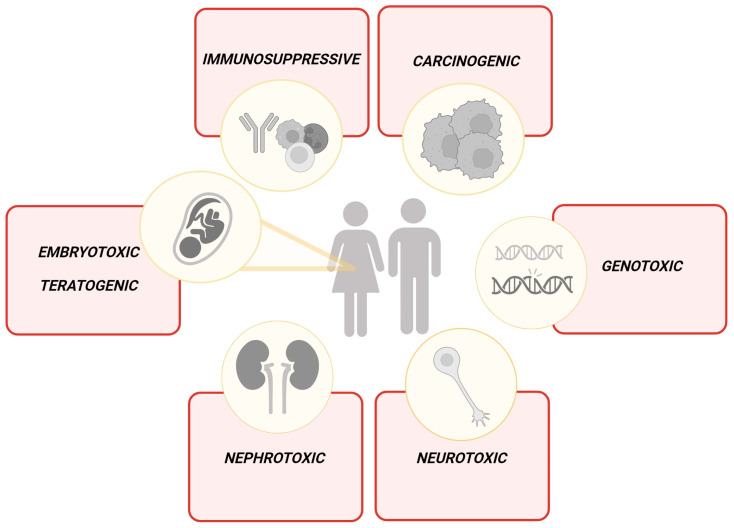
Multidirectional toxic effects of OTA on humans. Created in BioRender. Bijak, M. (2024) https://BioRender.com/z72k143 (accessed on 13 September 2024).

OTA exhibits multidirectional and harmful effects (Figure 1), including embryotoxic, teratogenic, genotoxic, neurotoxic, nephrotoxic, and immunosuppressive properties [26]. In 1987, the International Agency for Research on Cancer (IARC) classified OTA as a group 3 substance, indicating that it was not classifiable as carcinogenic to humans due to insufficient evidence [27]. However, six years later IARC reclassified OTA into group 2B, suggesting it is possibly carcinogenic to humans [28]. The reclassification was based on studies conducted on animal models, although direct evidence of OTA’s carcinogenicity in humans remains insufficient [29]. In a study by Al-Redha et al., a significant association was observed between cancer risk and the presence of OTA in the blood. The study cohort consisted of oncology patients, while the control group comprised healthy volunteers. The finding revealed that 60% of the oncology patients exhibited detectable levels of OTA in their blood, compared to only 12% in the control group. The highest rates of OTA contamination were found in patients aged 50–63 years and 64–77 years, with both groups showing a contamination rate of 33.3%. Furthermore, 60% of the blood samples from male patients were contaminated with OTA [30]. In response to dietary exposure, recent research has been undertaken to examine the presence of OTA in food and its potential impact on human health across various countries. Kouadio [31] analyzed mycotoxin contamination, including OTA, in various food products in Côte d’Ivoire. Based on the obtained data, the Estimated Daily Intake (EDI) of OTA was higher than the Tolerable Daily Intake (TDI) as recommended by the Joint FAO/WHO Experts Committee on Food Additives. Furthermore, the cancer risk was estimated by two methods—Threshold of Toxicological Concern (TCC) and Margin of Exposure (MOE). Following the TCC approach, an EDI concentration above 2.5 ng/kg bw/day suggests a risk of cancer for the population. As reported, EDI values for OTA in rice, maize, attieke, and peanut were 0.74–69, 0.4–397, 0.–12.8, and 0.2–58 ng/kg bw/day, respectively. The calculated MOE value, the ratio between a toxicological threshold derived from animal studies and the estimated human exposure [32], indicated that neoplastic effects could occur after OTA exposure in extreme concentrations as opposed to minimum concentrations [31]. Other studies also report OTA contamination of various food products, indicating a high risk of cancer, similarly based on MOE values [32,33]. However, some studies suggest the opposite effect following low OTA contamination, and therefore its lower consumption through the diet. In the study conducted by Hassan et al., OTA was found in 12 out of 38 tested samples, with only three samples reaching a concentration higher than the Lebanese and European limits. As reported, MOE values did not reach alarming levels, as they were >10,000 and >200 for the non-neoplastic and neoplastic effects, respectively [34]. Similar results were obtained by Oztekin and Karbancioglu-Guler, where the MOE value for OTA reached over 10,000, suggesting no health risk due to dried red pepper consumption [35]. In addition, Capei et al. reported no significant cancer risk associated with the consumption of OTA-contaminated food as the mean EDI ranged from 2.9% to 8.6 of the TDI recommended by the European Food Safety Authority (EFSA) when calculated based on the total diet [36]. Due to discrepancies, varying levels of contamination in different food products and differences in exposure to toxins due to regional dietary habits should be considered. In addition, in 2020, EFSA [37] published a report questioning the use of MOE as an effective method for determining the risk of cancer, which is related to the still unclear mechanisms of action of OTA. However, until research conducted in this direction fills the existing gap, this method remains in use. Moreover, EFSA indicated a strong need to continue other studies on OTA, such as on its occurrence [37].

### 2.1. Urinary System

#### 2.1.1. Studies Determining the Occurrence of Cancer in OTA Treatment

Urinary tract cancers, as classified by the International Statistical Classification of Diseases and Related Health Problems (ICD-10), encompass a range of malignancies affecting the kidneys, renal pelvis, ureter, bladder, and other unspecified organs [38]. Renal cell carcinoma (RCC) constitutes 2–3% of all malignancies, with the highest prevalence observed in Western Europe and the United States. Over the past two decades, the incidence of RCC has increased by approximately 2% annually both worldwide and in Europe. This rise is partly attributed to the enhanced utilization of imaging tests conducted for unrelated medical reasons, leading to increased detection of incidental lesions [39,40].

RCC is the predominant form of renal malignancy, accounting for approximately 90% of all kidney cancers. The majority of patients are male, with a male-to-female ratio of 1.5:1, and the peak incidence occurs between the ages of 60 and 70 [40]. The etiology of kidney cancer remains not fully elucidated. Most cases of kidney cancer are sporadic, while about 4% are familial, with some linked to genetic conditions such as von Hippel-Lindau (VHL) syndrome [41]. Additionally, it is hypothesized that exposure to food contaminants like mycotoxins, particularly ochratoxin A, may elevate the risk of developing urinary tract cancer [42]. The nephrotoxic effects of OTA have been well-documented, with several different mechanisms proposed to be involved in this process, including pyroptosis, organic anion membrane transporters, lipotoxicity, the ubiquitin–proteasome system, and autophagy [43].

The initial documentation regarding the impact of OTA on renal cancer development emerged in the 1980s. Two independent investigations demonstrated that administration of OTA via gastric intubation induced the formation of RCC. Bendele et al. observed that male B6C3F1 mice developed renal carcinomas and adenomas when exposed to OTA at a concentration of 40 ppm [44]. In turn, the 1989 report by the National Toxicology Program found that administering OTA by gavage for 9 or 15 months to F344/N rats was associated with an increased incidence of renal tubular cell tumors in males, as well as severe nephrotoxic effects [45].

Early studies performed on laboratory rats and mice administered OTA showed variable results. In a study conducted by Purchase and van der Watt in 1971, oral intake OTA was shown to be not carcinogenic in rats [46]. Kanisawa and Susuki in 1978 showed that three of nine mice administered OTA developed renal cell tumors. In comparison, no tumors were found in the control group [47]. Similar results were presented in Kanisawa et al.’s study in 1984, in which renal adenomas, carcinomas, and preneoplastic kidney lesions were observed in animals administered OTA; however, control animals were free of tumors [48].

In the abovementioned studies, there are a few limitations that should be considered when concluding. Lack of statistical analysis, short time of OTA exposure (less than 1 year), and inadequate administered doses that are not sufficient to evaluate the relationship between OTA and the presence of kidney tumors. Thus, more detailed studies were necessary to evaluate the role of OTA in carcinogenicity.

In the study conducted by Stoev, specific pathogen-free chicks were divided into two groups. The first group was administered OTA (5 mg/kg) and the second group received OTA (5 mg/kg) and L-β-phenylalanine (PHE) (25 mg/kg) for 24 months. One chick was dead at the end of the 10th month, while four chicks died between the 12th and 24th months. Results showed that chicks in the first group developed lymphosarcoma in the kidney and carcinoma in the part of the ureters, while chicks in the second group developed adenocarcinoma in the kidney. Statistical analysis showed a significantly augmented number of tumor incidents in comparison to the control group. Furthermore, PHE, which was shown to have a protective effect against OTA, showed no statistical significance between the studied groups. OTA administration also caused shrinkage and size reduction of kidneys. Pathomorphological examination of the kidneys revealed degenerative changes, such as granular or hydropic degeneration, mononuclear cell proliferation in the ureter mucosa, the proliferation of connective tissue, and mononuclear cells in the renal interstitium. Both groups also exhibited varying degrees of congestion in the peritubular capillaries, edema, proliferation, and activation of adventitial cells and endothelium (capillary and vascular). Moreover, infiltrative and destructive growth of secondary metastases with their origin in ureters and large necroses in the neoplastic tissue were also found in kidneys [38].

Mantle et al. performed a study in which OTA (3 mg/kg at the beginning, 1 mg/rat after animals grown) was administered to male Fischer-344 rats for 24 months in daily diet. Histopathological examination showed that among renal tumors, most of them were identified as unilateral carcinomas and the total percentage of incidence reached 25%. In the comparison, there was no renal tumor incident in the control group [39]. Kidney adenoma was also found in Fischer-344 rats administered OTA in a study performed by Mantle et al. [40].

In a study conducted by Stoev et al., BALB/c albino mice were administered OTA for 20 months. The authors divided 120 mice into four groups (three experimental and one control) in which animals received OTA and/or penicillic acid (PA). The feed was supplemented with OTA (approximately 1.4 mg/kg) and/or PA (approximately 7–8.4 mg/kg) and given to animals ad libitum. Histopathology examination showed that kidneys in mice treated with OTA revealed granular, vacuolar, and hyaline degeneration mainly in the proximal tubules after 14 days of administration. After 3 months of OTA exposure, mononuclear cell and connective tissue proliferation with simultaneous partial atrophy of some tubules was confirmed. Furthermore, polyploidy, aneuploidy, karyomegaly, small necrotic foci, swollen or occluded tubular lumen, and even small hemorrhages were also observed in mice treated with OTA. Additionally, changes were more severe in the group administered OTA + PA. Neoplastic changes in kidneys were found in eight mice (five benign neoplasia and three malignant neoplasia) from both OTA-treated groups [41].

In a study performed by Son et al., C57B/6N male mice were administered various concentrations of OTA (0.5–8 mg OTA/kg). Results showed that after 7 days of oral administration, higher concentrations of OTA (2 mg–8 mg OTA/kg) caused a significant decrease in body weight of mice in comparison to the control group. Histopathological changes were observed in the group treated with 1 mg/kg and above. Microscopic analysis showed significant necrosis of tubular cells. Moreover, serum biomarkers (creatinine, BUN) were also elevated after OTA treatment. OTA administration also induced kidney apoptosis in a dose- and time-dependent manner, led to necroptosis, induced oxidative stress, dysregulated mitochondrial homeostasis, and stimulated ferroptosis [49].

#### 2.1.2. The Potential Mechanisms of Action

The potential for OA-related carcinogenicity in humans has been explored through studies on the etiology of Balkan Endemic Nephropathy (BEN). The association between OTA, urothelial tumors, and BEN has not yet been determined; however, a high incidence of carcinoma in the bladder, kidney, and ureters and elevated concentrations of OTA in patients with BEN has been confirmed [50,51,52].

The mechanism of OTA’s carcinogenic action is not yet fully understood; however, several potential hypotheses have been proposed to explain this phenomenon. One of them is excessive lipid peroxidation, which has been demonstrated both in vitro and in vivo following OTA exposure [53,54,55,56,57,58]. The result of increased lipid peroxidation is the formation of malondialdehyde (MDA), which has mutagenic and carcinogenic properties [59]. The link between kidney cancer and lipid peroxidation has been demonstrated in several in vivo cancer models. One example is a comparison of the effects of two compounds: iron nitrilotriacetate (Fe-NTA) and aluminum nitrilotriacetate (Al-NTA). The former exhibits strong carcinogenic properties, unlike the latter. After intraperitoneal administration of both chemicals, rats receiving Fe-NTA showed a significant increase in lipid peroxidation products (e.g., MDA) and DNA adducts, compared to rats receiving Al-NTA and those in the control group that did not receive any compounds [59]. Similar results were obtained in the study conducted by Liu et al. in which another ferric iron chelate, Fe-EDDA, was evaluated [60]. Another example of lipid peroxidation and renal cell tumors was shown with potassium bromate [61].

Another mechanism responsible for indirect carcinogenesis in kidney cells is the ability of OTA to cause oxidative stress, and oxidative stress-induced apoptosis [58,62]. ASK1 belongs to the Mitogen-Activated Protein (MAP) kinase kinase kinase family and plays a crucial role in oxidative stress-mediated cell death. In research by Liang et al., it was shown that OTA triggers ASK1 activation in the human embryonic kidney cell line (HEK293), resulting in elevated reactive oxygen species (ROS) production and a reduction in mitochondrial membrane potential. Additionally, proteomic analysis indicated that a 24-h OTA exposure alters pathways associated with mRNA splicing, nucleotide metabolism, the cell cycle, lipid and lipoprotein metabolism, and DNA repair, processes in which ASK1 plays a critical role [62].

The hypothetical mechanism of action proposed by the authors suggests that OTA, which has a documented ability to generate ROS, induces the activation of ASK1, which in turn amplifies the excessive ROS synthesis. Furthermore, the activation of ASK1 in conjunction with OTA leads to DNA damage through reduced deoxyribonucleotide biosynthesis (genes RPM1, RPM2), decreased purine and pyrimidine metabolism (POLA1, POLR2A, and POLR2B), and diminished mitochondrial thymidylate biosynthesis. DNA damage thus occurs through three pathways, affecting cell cycle arrest, RNA synthesis, and DNA repair. (1) In the case of cell cycle arrest, reduced expression of proteins regulating the cell cycle was observed, particularly those involved in G2/M progression (CDC5L, CDK1), S-phase replication (POLA1, PCNA, RRM1, RRM2, TYMS, and MCMBP), as well as the formation of a bipolar spindle during mitosis (KIF11). (2) OTA in cells with activated ASK1 also led to decreased expression of proteins associated with the spliceosome (EIF4A3, DDX39B, CDCL5, XAB2, PRPF19, HNRNPL). (3) Furthermore, the study demonstrated that OTA down-regulates six key proteins involved in DNA repair, including those essential for nucleotide excision repair (POLR2A, POLR2B, and PCNA), transcription-coupled repair (XAB2), and DNA double-strand break repair (PRPF18). The down-regulation of these proteins suggests that OTA-induced DNA damage may be partly due to an impaired DNA repair system. Additionally, ASK1 knockdown reversed (POLR2B, XAB2) or alleviated (CDK1, PCNA) the down-regulation of several of these proteins, indicating that ASK1 may play an inhibitory role in DNA repair processes [62].

Carcinogenesis may result from mutagenesis due to oxidative damage in DNA caused by OTA [29,63]. Kamp et al. conducted a study in which male F344 rats were administered OTA in different concentrations daily for four weeks. The results obtained, using the comet method with the use of the repair enzyme formamido-pyrimidine-DNA-glycosylase (FPG), indicated OTA-mediated oxidative damage in DNA in the kidney. The toxin doses used in the study were within the range that caused kidney tumors in rats. Therefore, the research on oxidative DNA damage may help in understanding the mechanism of carcinogenesis [64].

The biotransformation of xenobiotic substances in the body, primarily facilitated by CYP enzymes during phase I reactions, can lead to the formation of carcinogens and subsequent oxidative stress, which damages cellular molecules. The activation of transcription factors such as aryl hydrocarbon receptor (AhR) and pregnane X receptor (PXR), which regulate the CYP450 enzymes (CYP1A2, CYP1A2, and CYP3A4), can further induce immunosuppression, cancer, and alter drug toxicity. In the study conducted by Lee et al., OTA caused kidney damage in HK-2 cells, which was evaluated based on the expression of kidney injury molecule-1. Furthermore, OTA increased generation of ROS, decreased glutathione (GSH) level, elevated concentrations of MDA, and upregulated the expression of phase I (AhR, PXR) and phase II enzymes (heme oxygenase-1 (HO-1), glutamate-cysteine ligase catalytic subunit (GCLC), NAD(P)H quinone dehydrogenase 1 (NQO1)) of xenobiotics metabolism in vitro and in vivo at the mRNA and protein level. Moreover, OTA resulted also in the upregulation of NF-E2-related factor 2 (Nrf2), which is a transcription factor responsible for the regulation of expression of phase II enzymes (i.e., HO-1, NQO1, GCLC), AhR, and PXR in HK-2 cells [65]. Results of the Lee et al. study showed that exposure of HK-2 cells to OTA led to the activation and translocation of nuclear xenobiotic receptors AhR and PXR, resulting in the induction of CYP1A1, 1A2, and 3A4 enzymes. Inhibition of AhR and PXR expression via siRNA reduced ROS production, indicating that ROS generation is mediated by AhR- and PXR-induced CYP enzymes in response to OTA. This suggests that OTA metabolism by CYP enzymes contributes to renal toxicity, with ROS playing a key role in kidney damage through AhR- and PXR-dependent pathways.

Another proposed mechanism is associated with the altered signaling pathways of MAPK and phosphatidylinositide 3-kinase (PI3K)/protein kinase B (Akt) as a result of OTA exposure. The extracellular-regulated kinase 1-2 (ERK1-2) pathway within the MAPK family possesses a dual role, which is traditionally associated with cell growth, proliferation, and differentiation, but can also promote apoptotic cell death under certain stress conditions. Inhibition of ERK1-2 has been linked to enhanced cell survival and reduced apoptosis. Additionally, the PI3K/Akt pathway is identified as a crucial regulator of cell survival and proliferation, with its hyperactivation often contributing to resistance to apoptosis and promoting malignant transformations, including in kidney cancer. This pathway plays a key role in balancing apoptosis by inhibiting pro-apoptotic proteins and is critical for cellular proliferation, survival, and metabolism [66]. OTA was shown to be associated with the activation of ERK-1-2 and MAPK signaling in renal cells [67,68]. In a study conducted by Özcan et al., OTA caused the activation of both: PI3K/Akt via c-MET, a receptor tyrosine kinase, and MEK/ERK1-2 pathways in the HK-2 cell line. Interestingly, both pathways act differentially by sustaining cell survival or promoting apoptosis, respectively. Moreover, the activity of executioner caspase-3/7 was also increased after the exposition of cells on OTA. Based on the results obtained from Özcan et al., apoptosis was shown to be stimulated and sustained through the MEK/ERK1-2 pathway [66]. Similar results were obtained in a study conducted by Darbuka et al.; however, authors also showed that OTA augments the DNA-binding activity of NF-ĸB and causes nuclear shuttling of NF-ĸB by translocation of p65 protein in the HK-2 cell line. The above-mentioned evidence may suggest that increased activation of NF-ĸB leads to both a substantial increase in ERK1/2 phosphorylation and the induction of apoptosis mediated by ERK1/2 phosphorylation after extended OTA exposure [69].

OTA-induced carcinogenesis may also be associated with the hypoxic environment. In a study performed by Raghubeer et al., OTA was shown to significantly alter the protein concentration of hypoxia-inducing factor 1 (HIF1α), heat shock protein 90 (HSP90), and pyruvate dehydrogenase kinase 1 (PDK1) in a time and dose-dependent manner in HEK293 cells. Furthermore, gene expression of vascular endothelial growth factor (VEGF), transforming growth factor β (TGFβ), and erythropoietin (EPO) also varied by time and dose. HIF1α and HSP90 collaborate closely in processes like apoptosis, induction of VEGF, angiogenesis, stabilization of protein conformation, and carcinogenesis, i.e., by increasing cancer cell survival, augmenting proliferation, and stabilizing adjustment to harsh conditions [70]. The evidence confirming the association between OTA and hypoxic response in human kidney cell lines and in mice was also obtained by Pyo et al. [71] and Yang et al. [72].

Renal carcinogenesis caused by OTA may be a combination of genetic variability and augmented proliferation that leads to mitotic disruption. In a study conducted by Mally [73], a potential mechanism of carcinogenicity was suggested, proposing that multiple factors like OTA uptake, inhibition of histone acetyltransferases (HATs), disruption of mitosis, cell proliferation, and genetic instability act simultaneously. In a Czakai et al. study [74], OTA was shown to inhibit the total activity of HATs from human kidney epithelial cells, thus altering the acetylation of histone and nonhistone proteins associated with the wide range of biological processes affected by OTA, including DNA damage and repair, chromatin condensation, gene transcription and regulation of its factors’ activity. In a study conducted by Rached et al. [75], OTA was shown to block mitosis at the metaphase–anaphase transition in human kidney epithelial cells. Furthermore, the results showed that OTA activates pro- and antiapoptotic signaling pathways leading to cell death. These findings agree with the results of studies that demonstrated the G2/M cell-cycle arrest caused by OTA [62,76,77,78]. In vivo, OTA-induced morphological changes linked to mitotic dysfunction have been observed across different treatment conditions, aligning with the site-specific formation of renal tumors caused by OTA [73]. Compensatory cell proliferation following direct or indirect cytotoxicity is recognized as a key factor in the carcinogenicity of various renal carcinogens operating through a nongenotoxic mechanism of action [79]. In vivo studies consistently show target- and site-specific increases in cell proliferation in response to OTA-induced cell loss, occurring in a time- and/or dose-dependent manner. Notably, this increase in renal tubular cell proliferation is more pronounced in male rats, correlating with their greater susceptibility to tumor development compared to females [75,80].

**Figure 2:**
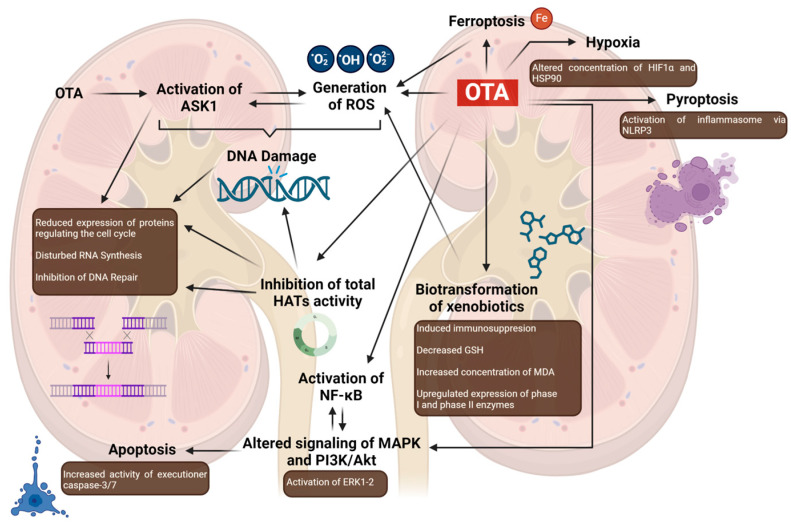
Mechanisms of OTA-induced nephrotoxicity. Abbreviations: Akt: protein kinase B; ERK1-2: extracellular-regulated kinase 1–2; GSH: glutathione; HATs: histone acetyltransferases; HIF1α: hypoxia-inducing factor 1; HSP90: heat shock protein 90; MAPK: Mitogen-Activated Protein Kinase; MDA: malondialdehyde; NF-ĸB: nuclear factor kappa B; NLRP3: (NOD)-like receptor family pyrin domain containing 3; OTA: ochratoxin A; PI3K: phosphatidylinositide 3-kinase; ROS: reactive oxygen species. Created in BioRender. Bijak, M. (2024) https://BioRender.com/w05z826 (accessed on 12 October 2024).

Ferroptosis is a new type of non-apoptotic cell death, first proposed by Stockwell and Dixon. This form of cell death is characterized by the massive accumulation of iron and excessive generation of ROS [81]. A study conducted by Wang et al. showed that OTA induced ferroptosis in HEK-293 and HK-2 cells. Upon treatment, altered morphological changes, augmented iron concentration, and increased levels of lipid ROS were observed in both cell lines [82]. Similar results confirming the association between OTA and ferroptosis were obtained in other studies focused on kidney cells [49,83,84,85]. Ferroptosis is closely related to tumor biology, due to its potential suppressing role. Cancer cells typically exhibit resistance to apoptosis; however, they exhibit susceptibility to ferroptosis [86]. The ability of OTA to induce ferroptosis presents intriguing prospects for the targeted use of this metabolite in medicine. Pyroptosis is a type of programmed cell death caused by the response of the inflammatory system as an effect of microbial infection of host factors. In recent years, studies have shown that mycotoxins may stimulate pyroptosis in various cell types. A detailed mechanism of pyroptosis was described in the study conducted by Liao et al. and is strictly related to the (NOD)-like receptor (NLR) family pyrin domain containing 3 (NLRP3) protein, which is a crucial component of inflammasomes [87]. Few studies have focused on the interaction between OTA and NLRP3 inflammasome-mediated pyroptosis. Research performed on Madin–Darby canine kidney cells showed significant activation of NLRP3 inflammasome, overexpression of pro-inflammatory cytokines (i.e., interleukin 1β (IL-1β), IL-6, IL-18, tumor necrosis factor α (TNFα)), and activation of caspase-1 [88]. OTA also induced pyroptosis in a pig kidney cell line (PK-15), contributing to nephrotoxicity by upregulating NLRP3 and associated proteins [89]. In an in vitro study, Li et al. demonstrated that OTA-triggered nephrotoxicity is linked to NLRP3 inflammasome-mediated pyroptosis in PK-15 cells. Moreover, OTA significantly increases the expression of pro-inflammatory cytokines, including TNF-α, IL-1β, IL-18, and IL-6 [90]. The above mechanisms are presented in Figure 2.

Bioactivation and subsequent DNA adduction through covalent attachment of electrophilic OTA species remain a viable mechanism for OTA-mediated carcinogenesis [63]. Pfohl-Leszkowicz et al. conducted a study in which three different organs, including the kidney, were examined for the occurrence of DNA adducts formed as a result of OTA exposure. The researchers detected the formation of 24 adducts, but the rate of their occurrence was time-dependent. Some of them appeared after 8 h, while others were detected after 24, 48, 72, or 120 h. Moreover, differences in their stability were also noted—seven adducts could be detected after 16 days [91]. Mantle et al. conducted an in vivo study and administered OTA to 8-week-old male Fischer and Dark Agouti rats for three days. As the researchers reported, a refined 32P-post labeling methodology was used, and as a result, one principal adduct isolated in small amounts from the kidneys of rats was detected—ochratoxin B-guanine. Moreover, the authors of the publication drew attention to an inaccuracy in the preparation of the protocols by previous researchers, which could negatively influence the results indicating the lack of a genotoxic effect of OTA [92]. Another study conducted by Petkova-Bocharova et al. demonstrated the presence of DNA adducts in OTA-treated pigs, while no animals in the control group exhibited these adducts. In addition, researchers observed differences between the sexes of the studied animals in the case of different expressions of CYP450 due to different expressions of ECOS, PROD, and EROD enzymes. Moreover, sex was also associated with differences in the modulation of cyclooxygenase (COX) pathways by OTA [93]. Castegarno et al. conducted a study on DA and Lewis rats of both sexes, pointing out the differences between the animals post-OTA administration. As reported, the most sensitive for OTA-induced renal adenocarcinoma were DA male rats, whereas DA females were resistant. In the case of Lewis rats, a moderate response was observed. Furthermore, the researchers found DNA adducts in the kidneys that were significantly associated with renal carcinogenicity. The results revealed differences between the sexes of DA rats, with male rats exhibiting the highest levels of adducts while female rats displayed the lowest levels. OTA administration resulted in a significant increase in the number of karyomegalies in the kidneys of the studied rats, but not in DA females. These changes were accompanied by the development of kidney tumors [94].

Brown et al. conducted a study on male Fischer F-344 rats. The animals were divided into groups and administered OTA daily for two years. The analytical findings substantiated a marked change in DNA ploidy. The distribution was diploid in all renal adenomas and aneuploid in all carcinomas, correlating with their typical organized and disorganized histopathology, respectively. Aneuploidy was also detected in renal tissue [95].

MicroRNA (miRNA) is known for its role in physiological and development processes but also as a crucial cause of cancer development and progression, as it controls the proliferation of cells and apoptosis [96]. A study conducted by Zhao et al. focused on high-throughput miRNA profiling and KEGG bioinformatic analysis in HEK293 cells. The cell culture was treated with 25 µM OTA for 24 h and after incubation, the researchers observed cell apoptosis. The viability and mitochondrial membrane potential (Δψm) of HEK293 were significantly decreased, which could be due to ROS generation, inhibition of protein synthesis, and induction of stress response. As reported, OTA induced deregulation of a mass of miRNA. The study also used liver cells (HepG2) to make comparisons between lines. Zhao and coworkers observed disturbances in miRNA synthesis due to the suppression of DGCR8, Dicer1, and Drosha in HEK293 cells. Moreover, the researchers reported upregulation of both miR-122 and miR-4289, and a decrease in miR-125b and miR-378d expression. In addition, numerous miRNAs within the commonly expressed miRNA groups (CEG) were associated with transduction pathways and the deregulated miRNAs within the differentially expressed miRNA groups (DEG) exhibited a stronger relevance to human cancer, including significantly increased miR-184 and miR-7-5p, respectively. Additionally, a significant decrease in c-Myc was observed [96].

### 2.2. Gastrointestinal System

#### 2.2.1. Liver Cancer

According to recent global cancer statistics, there were 865,269 new cases of liver cancer in 2022, accounting for 4.3% of all new cases and ranking it sixth in terms of incidence. Additionally, 757,948 new deaths were reported, placing liver cancer in third place [97]. Liver cancer is a general term for a variety of histologically diverse primary tumors of the liver and bile duct. The most common type, accounting for more than 80% of all cases, is hepatocellular carcinoma (HCC) [98]. HCC exhibits diverse epidemiological characteristics, including temporal fluctuations, regional and demographic disparities, and environmental risk factors. Depending on the region, other major risk factors for HCC are distinguished [99].

OTA is implicated in liver toxicity, a phenomenon extensively documented in numerous studies [100,101,102,103,104,105,106]. Furthermore, several investigations have suggested a potential correlation between OTA exposure and the incidence of liver cancer. Imaida et al. performed a study using rats whose diet included various mycotoxins, including OTA. Administering OTA in either the initiating or the promoting stage significantly increased the induction of hyperplastic nodules. Moreover, OTA was observed to substantially enhance both the size and number of these nodules [107]. In turn, Bendele et al. performed a study on weanling male and female mice, administering OTA at two concentrations, 1 and 40 ppm, over a 24-month period. As reported, HCC incidence increased at 40 ppm in both sexes. In the 1 ppm group, an increase was observed only in males. However, statistical significance solely in the case of female mice was observed. Furthermore, the researchers observed hepatocellular adenoma occurrence that concerned male mice administered both OTA concentrations [44]. Stoev, in a study mentioned in the previous section, reported various types of neoplasia in the liver. The researcher mentioned one adenocarcinoma and one adenoma in the OTA-fed group, while no neoplastic changes in the control group were found [41]. In an earlier study by the same researcher, malignant adenocarcinoma was observed in male chicks fed with 5 ppm-mg/kg of OTA, while also found in female chicks fed with 5 ppm-mg/kg of OTA combined with PHE [38]. Hyperplastic nodules and hepatic cell tumors were detected in the livers of mice administered OTA and/or aflatoxin. When animals were administered 40 ppm of OTA one hyperplastic nodule and five hepatic cell tumors emerged. In the group administered DMSO + OTA, minor changes were observed, since two and three hyperplastic nodules and hepatic cells emerged, respectively. Therefore, OTA did not cause many neoplastic changes; however, when combined with aflatoxin both toxins acted synergistically, enhancing the hepatotoxic effect [47]. The study conducted by Kanisawa and Suzuki indicated that OTA has carcinogenic potential in the mice’s livers. This risk may be exacerbated when OTA is combined with other hepatotoxic and cancerogenic factors.

Kortei et al. reported high levels of aflatoxin and OTA contamination in maize in Ghana. More than half of the tested samples exceeded the levels set by the EFSA and the local agency, Ghana Standards Authority (GSA). In addition, studied maize *Zea mays* L is a principal cereal, extensively consumed, and accounts for 40% of the cereal production in Sub-Saharan Africa, with over 80% used as food. Due to its high demand and consumption, residents are incessantly exposed to both mycotoxins [108] and, because both are hepatotoxic when combined, they can pose a serious health risk.

Ibrahim et al. tested serum samples from HCC patients for the presence of OTA and to quantify its association. As reported, the highest incidence of elevated OTA was found in the HCC group and was 5-fold higher in comparison to the control group. The concentration of OTA ranged between 0.129 and 10.93 ng/mL with a mean value of 1.1 ± 0.3 ng/mL, and 0.005 and 0.50 ng/mL with a mean value of 0.201 ± 0.02 ng/mL in the HCC group and control group, respectively [109]. Another study also focused on the relationship between HCC and OTA presence. The incidence of HCC was higher among 29 patients with liver cirrhosis who tested positive for OTA compared to those with a negative result, although the overall results did not exhibit this relationship. Therefore, OTA may be a risk factor for cancer in cases of an already damaged liver [110]. Among mycotoxins, HCC is usually associated with aflatoxin [111]. However, both mentioned studies suggest it may also be linked to OTA while highlighting the necessity for further research.

HCC is known as one of the most deadly malignancies and its development and progression may be related to chromosomal aberrations, epigenetic abnormality, and changes in gene expression [109]. Multiple genetic events have been linked to the development of HCC. These include the inactivation of the tumor suppressor p53, mutations in β-catenin, overexpression of ErbB receptor family members, and overexpression of the MET receptor. Additionally, certain cancer-related genes appear to be targeted by epigenetic mechanisms, such as methylation, in human HCC. It is believed that various processes, including telomere erosion, chromosome segregation defects, and alterations in DNA damage response pathways, contribute to these mechanisms [98].

Bouaziz et al. proposed a potential pro-carcinogenic mechanism for OTA, demonstrating that OTA induces a caspase-dependent mitochondrial apoptotic pathway in HepG2 cells. The mitochondrial alterations include bax relocalization into the mitochondrial outer membrane, loss of Δψm, permeability transition pore complex (PTPC) opening, and cytochrome c (but not apoptosis-inducing factor (AIF)) release. Furthermore, OTA induced O_2_^−^ generation, which appears to be a consequence of mitochondrial alterations. HepG2 cell treatment with the p53 inhibitor pifithrin- (PFT) and Western blot analysis suggested that OTA triggers a p53-dependent apoptotic pathway [112]. HepG2 cells were also studied by Zhao et al. for high-throughput miRNA profiling [96] as previously mentioned in comparison with HEK293 cells. The PCR result showed the down-regulation of DGCR8, Drosha, and Dicer1 in HepG2 cells. Moreover, among the four significantly altered miRNAs selected for RT-PCR measurement, miR-125b was notably down-regulated in OTA-treated HepG2, while the others, miR-122, miR-4289, and miR-378d, did not show significant changes. During CEG and DEG analysis, the researchers observed a significant increase in miR-183 in CEG and nearly the same in miR-7-5p in DEG. In the HepG2 cell line, CEGs were predominantly associated with signal transduction pathways, whereas DEGs were mostly related to human cancer pathways, a pattern also observed in the HEK293 cell line [96]. Zhu et al. conducted in vivo and in vitro studies focusing on miR-122 to investigate its role in OTA-induced hepatotoxicity. As reported, the results indicated that miR-122 was expressed differently in the livers of male rats than in HepG2 cells. Compared to the control, after four weeks of OTA administration, the expression of miR-122 was down-regulated, whereas after 13 weeks the effect was the opposite. In the case of HepG2 cells the researchers observed significantly increased expression after 20 µM OTA treatment [113].

**Figure 3:**
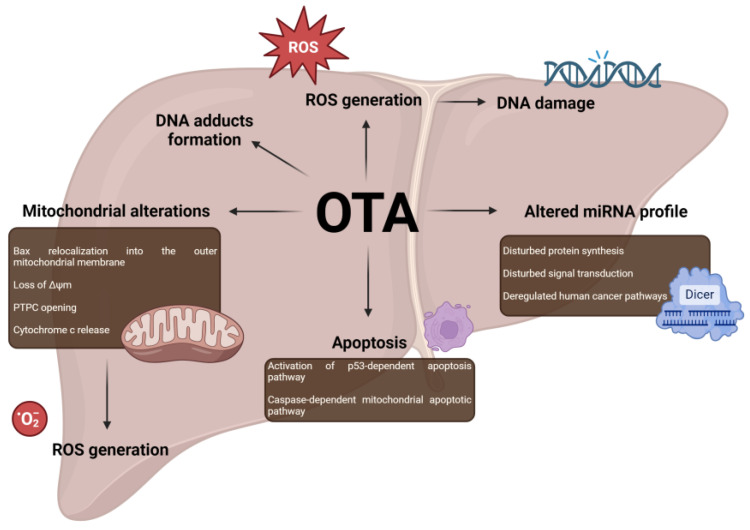
Mechanisms of OTA-induced hepatotoxicity. Abbreviations: OTA: ochratoxin A; PTPC: permeability transition pore complex; ROS: reactive oxygen species; Δψm: mitochondrial membrane potential. Created in BioRender. Bijak, M. (2024) https://BioRender.com/j83v757 (accessed on 13 October 2024).

Aflatoxin carcinogenicity in the liver arises from DNA damage, which includes the formation of DNA adducts and oxidative DNA damage [114]. OTA exhibits similar mechanisms. Pfohl-Leszkowicz et al. detected 42 DNA adducts per 109 nucleotides in the liver. Additionally, 15 DNA adducts were observed in the liver, with 11 of these potentially being common to those in the kidney. Notably, one of the adducts, which was the most stable in the kidney, remained in the liver for up to 72 h [91]. Furthermore, the study by Kamp et al. [64], as mentioned in the previous section, also demonstrated that OTA induces oxidative DNA damage in the liver of rats. The mechanisms of OTA-induced hepatotoxicity are presented in the Figure 3.

The research results undoubtedly indicate a link between hepatotoxicity and carcinogenesis associated with OTA. However, while the liver is particularly vulnerable, it is not the only organ in the gastrointestinal system where the toxic effects of OTA can be observed.

#### 2.2.2. Other Organs

Ikoma et al. suggested a potential link between OTA contamination in red chili peppers and gallbladder cancer (GBC). The researchers collected red chili peppers from Peru, Chile, and Bolivia, which are countries with high incidences of GBC, and assessed OTA concentration using high-performance liquid chromatography. The study showed that OTA levels in some samples exceeded the limits recommended by the European Commission. However, while Ikoma et al. did not establish a definitive link between OTA contamination and GBC, they highlighted the potential association and emphasized the need for further research [115].

Gastrointestinal cancers include esophageal, gastric, colon, and rectal tumors [116]. Bray et al. [97] reported new cases of colorectal, stomach, and esophagus cancers constituting 9.6%, 4.9%, and 2.6% of all cases, respectively, according to global cancer statistics. Colorectal cancer was thus classified as the third most common cancer, following lung and breast cancer, with 1,926,118 cases. It also had the second highest mortality rate, after lung cancer, with 903,859 cases [97].

Liu et al. performed a study on human esophageal epithelium immortalized cells (Het-1A) suggesting OTA toxicity associated with apoptosis with caspase-3 activation, DNA strand breaks, and chromosome aberrations. As reported, DNA damage was related to G2 cell cycle arrest and the down-regulation of Cdc2 and cyclinB1. Moreover, the authors mentioned possible relations to an early event initiating human esophageal cancer [117]. Zhao et al. used the same cell line in their study and observed several effects following OTA exposure. The researchers noted decreased cell viability and increased apoptosis-related indices, including ROS generation, oxidative DNA damage, mitochondrial dysfunction, reduced Δψm, release of cytochrome c, and mitochondrial apoptotic pathway activation. Additionally, the study examined glucose metabolism, revealing that OTA shifted glucose metabolism in Het-1A cells from oxidative phosphorylation to glycolysis. This shift was associated with a decrease in the expression of tricarboxylic acid (TCA) cycle enzymes such as α-ketoglutarate dehydrogenase (OGDH) and isocitrate dehydrogenase 1 (IDH1), revealing that OTA shifted pyruvate dehydrogenase kinase 1 expression. The results indicated significantly elevated glucose consumption and lactate production in cells exposed to 10 µM OTA. These findings underscore the potential of glucose metabolism as a metric for assessing the cytotoxicity and carcinogenicity of OTA and contributing to a better understanding of the molecular mechanism underlying OTA-induced cytotoxicity in Het-1A cells [118].

**Figure 4:**
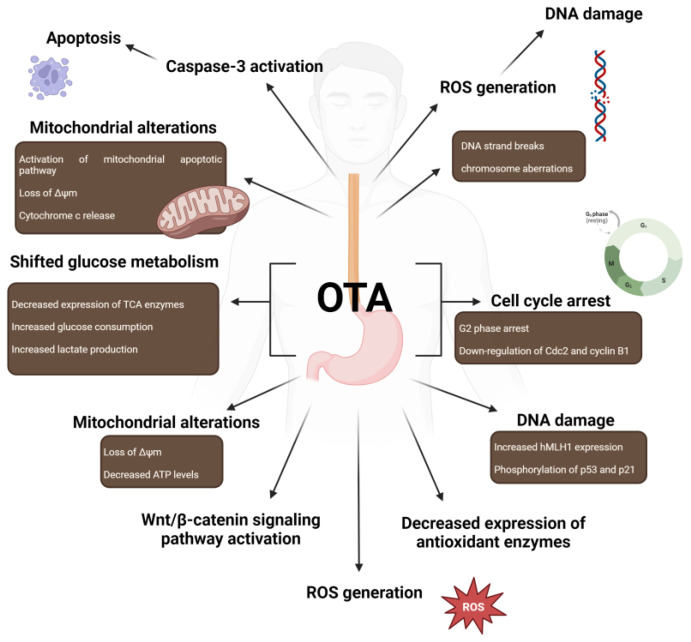
Mechanisms of action of OTA in esophagus and stomach. Abbreviations: OTA: Ochratoxin A; ROS: Reactive Oxygen Species; TCA: tricarboxylic acid; Δψm: mitochondrial membrane potential. Created in BioRender. Bijak, M. (2024) https://BioRender.com/n17b071 (accessed on 5 October 2024).

In various studies, researchers have focused on human gastric epithelium cells (GES-1). As reported, OTA can induce cell cycle phase arrest [119,120]. In a study performed by Cui et al., OTA induced G2/M phase arrest with a major influence on the G2 phase. Moreover, the researchers reported reduced expression of key factors critical to G2/M phase transmission, including Cdc25C, Cdc2, and cyclinB, as a result of which apoptosis was detected [119]. In addition, OTA exposure could induce genomic instability and DNA damage. The OTA-induced DNA damage in GES-1 cells was related to the hMLH1-p53-p21 signaling pathway by increasing hMLH1 expression and phosphorylation of two proteins—p53 and p21 [120]. Jia et al. reported increased proliferation, migration, and invasion abilities in the same cell line. After 40 weeks of OTA treatment, GES-1 cells were injected into Balb/c nude mice for xenograft tumor observation. The obtained results suggest long-term exposition leads to increased ROS generation, activation of the Wnt/β-catenin signaling pathway, and consequently increased malignant transformation [121]. Another study on the GES-1 cell line showed similar effects from OTA as in the previously mentioned HET-1A cell line. Wang et al. reported a shift in glucose metabolism from oxidative phosphorylation to glycolysis, as well as an increase in glucose consumption and lactate production. Furthermore, the expressions of IDH1 and OGDH were decreased, whereas the expressions of other factors, including GLUT1 and glycolytic enzymes, were significantly increased [122]. Li and coworkers reported mitochondrial dysfunction after OTA treatment for 24 h. OTA presence resulted in increased ROS levels with decreased expression of antioxidant enzymes. Moreover, decreases in Δψm and ATP levels were reported. In addition, OTA toxicity led to apoptosis and autophagic cell death, as well as induced mitochondrial biogenesis [123]. The toxic effects of OTA on the esophagus and stomach are presented in the Figure 4.

Stoev also reported neoplasmic changes in other internal organs [41]. In the group administered OTA, only one lymphoma was found in the intestinal mesenterium. The control group did not show any neoplastic changes, which highlights the influence of OTA on their formation [41].

Abassi et al. conducted a study using human colon carcinoma cells (HCT116) and found that exposure to OTA significantly affected the clonogenic potential of this cell line, potentially through the upregulation of the c-myc gene’s transcriptome. Furthermore, OTA enhanced the migration of HCT116 cells at very low concentrations, suggesting that this mycotoxin may exhibit carcinogenic properties in these cells [124].

Based on the studies described, similarities can be observed in the mechanism of action of OTA and its toxicity on individual organs of the gastrointestinal system.

### 2.3. Rare Neoplasma

There is no uniform definition of rare malignant neoplasms. In the United States, cancers are considered rare if they occur at a frequency of less than 15 cases per 100,000 individuals. In contrast, in Europe, the threshold is fewer than six cases per 100,000 individuals [125,126]. A total of 186 disease entities have been identified as sources of particular challenges concerning clinical decision-making, healthcare organization, and clinical research due to their low incidence. Epidemiological differences exist between various countries [127]. All rare malignant neoplasms share common features related to diagnostic difficulties, linked to a lack of clinical practice and the absence of standardized treatment protocols and therapeutic guidelines. Rare cancers include sarcomas, which can affect both bone and soft tissues, tracheal tumors, middle ear tumors, nasal cavity and sinus cancers, neuroendocrine tumors, and ocular squamous cell carcinoma. Some extremely rare types occur with a frequency of less than one case per 100,000 individuals [128].

Ocular squamous cell carcinoma (OSCC) is the most common malignant tumor of the ocular surface. It is a rare disease, occurring at a frequency of 0.13–1.9 cases per 100,000 individuals, predominantly affecting individuals aged 50 to 70 years, with a higher prevalence in men. Squamous cell carcinoma of the conjunctiva represents the advanced stage of a condition known as ocular surface squamous neoplasia (OSSN) [129]. OSSN is a malignant ocular condition that can lead to vision loss and, in severe cases, mortality. The primary risk factors for both conditions include exposure to solar ultraviolet radiation, acquired and viral immunodeficiency syndromes (such as HIV/AIDS), human papillomavirus (HPV) types 16 and 18, and allergic conjunctivitis. In cases of immunodeficiency, the risk of developing conjunctival epithelial neoplasia is estimated to be 13-fold higher in patients with acquired immunodeficiency syndrome (AIDS) [130,131,132]. Soft tissue sarcomas, on the other hand, originate from mesodermal tissues. They are primarily located in the limbs (50% of cases), trunk (30%), head and neck (10%), and retroperitoneal space (10%). Due to factors such as delayed presentation for treatment after tumor observation, technically challenging surgical procedures, a high degree of malignancy in most cases, and a high tendency for local recurrence, the treatment outcomes for this group of tumors remain unsatisfactory. Soft tissue sarcomas account for less than 1% of all malignant tumors diagnosed in adults [133,134]. The etiology of soft tissue sarcomas is not yet well understood. However, these tumors occur more frequently in individuals with genetically transmitted diseases. Sarcomas have a higher incidence in individuals with conditions such as Recklinghausen’s disease (neurofibromatosis), Gardner’s syndrome, Werner’s syndrome, tuberous sclerosis, and Li–Fraumeni syndrome (p53 mutation) [135,136,137].

The influence of OTA on the development of rare cancers remains controversial due to the limited number of studies available. Nonetheless, it may be a significant observation in the context of the etiology of these diseases. It is noteworthy that the causes of many of these rare cancers are not precisely understood; thus, the investigation of new risk factors is warranted. Previous studies by Stoev demonstrated that rats exposed to 10 ppm of OTA in their feed developed squamous cell carcinoma of the conjunctiva, which infiltrated the cornea and was characterized by low differentiation [41,138]. Importantly, this type of cancer is extremely rare in rats, suggesting a direct correlation between its development and the consumption of OTA-contaminated feed [139]. Furthermore, these studies also identified the presence of sarcomas, including angiosarcomas (hemangioendothelioma), which exhibited clear malignant features such as low differentiation, polymorphism of neoplastic cells, and polychromasia. These findings underscore the potential carcinogenic effects of OTA and the necessity for further research to elucidate its role in the development of rare cancers [41].

**Figure 5:**
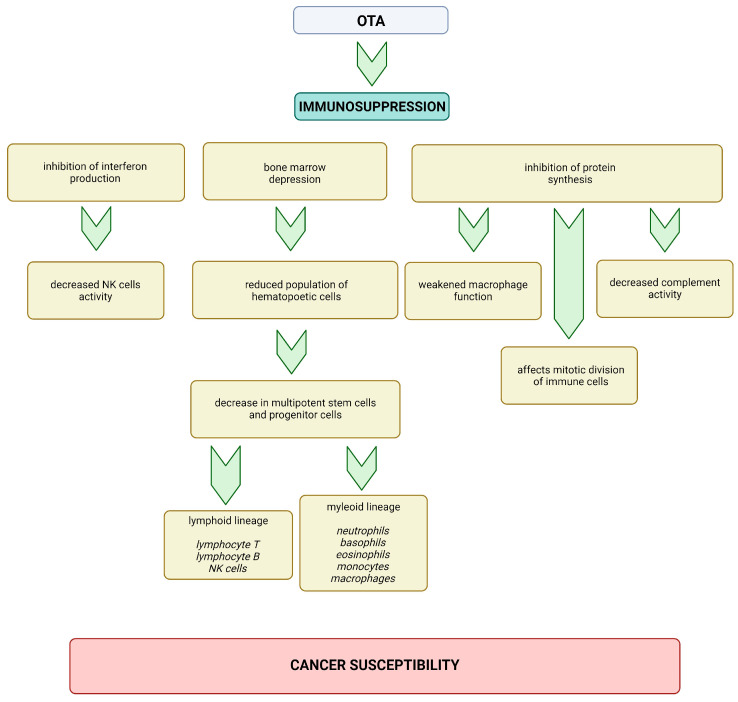
Potential mechanisms of OTA-induced carcinogenicity through immunosuppression. Created in BioRender. Bijak, M. (2024) https://BioRender.com/g46c679 (accessed on 13 September 2024).

One of the most significant factors predisposing individuals to the development of rare cancers, including squamous cell carcinoma (SCC), is immunosuppression. It is widely accepted that OTA exerts an immunotoxic effect. However, the precise mechanism underlying OTA’s immunosuppressive effects remains incompletely understood. Numerous studies suggest that OTA’s inhibition of protein synthesis adversely affects the mitotic division of rapidly dividing immune cells. This inhibition also diminishes complement activity and weakens macrophage function, which plays a critical role in the nonspecific immune response [37,140,141]. OTA-induced immunosuppression results in bone marrow depression, impairing both humoral and cellular immune responses, evidenced by a reduced population of hematopoietic cells [142]. The reduction in stem cell numbers initiates a cascade effect, leading to a subsequent decrease in multipotent stem cells and progenitor cells of various leukocyte fractions. This reduction affects both the lymphoid lineage (including T and B lymphocytes and NK cells), directly impacting the immune response, and the myeloid lineage (including neutrophils, basophils, eosinophils, monocytes, and macrophages) [143,144,145]. Another proposed mechanism by which OTA may contribute to carcinogenesis through immunosuppression is the inhibition of interferon production. This inhibition leads to decreased NK cell activity, further compromising the immune system’s ability to combat tumor cells. These multifaceted immunosuppressive effects of OTA underscore its potential role in increasing susceptibility to rare cancers [146,147,148]. The above mechanisms are presented in Figure 5.

Importantly, OTA exhibits immunosuppressive effects at high doses and long exposure times, while low doses and short exposure times activate the immune system [149]. Gan et al. found that OTA at a dose of 0.5–4 μg/mL could inhibit the proliferation of primary splenocytes in pigs [150]. Furthermore, Xu et al. showed that OTA at a dose of 0.5–1.5 μg/mL could increase the production of TNF-α and enhance the expression of the TLR4-MyD88-NF-κB signaling pathway [151]. In turn, Su et al. observed that exposure to OTA for more than 72 h increased the expression of anti-inflammatory cytokines, decreased phagocytosis and macrophage migration, and promoted the switching of macrophages from M1 to M2. Conversely, short exposure up to 24 h increased the expression of pro-inflammatory cytokines, enhanced migration and phagocytosis of macrophages, and promoted macrophage polarization to M1 [152].

## 3. Gender Differences

Gender differences in cancer incidence are well-documented. Renal cancer is diagnosed twice as often in men compared to women, with hepatocellular carcinoma showing similar trends [153,154,155]. Conversely, the incidence of malignant tumors in young adults (18–44 years old) is higher in women [156]. The underlying mechanisms driving these differences remain incompletely understood. It is hypothesized that sex hormones play a significant role, possibly in conjunction with the presence of the Y chromosome in men [157,158]. The immune system’s sensitivity to sex hormones appears to be of significant importance, with women exhibiting a stronger immune response. This heightened immune activity allows for more rapid pathogen elimination in women; however, it also increases their susceptibility to autoimmune reactions and inflammation, potentially predisposing them to cancer [159]. The higher incidence of OTA-related cancers in males may be attributable to the immunosuppressive effects of OTA, which, when coupled with the inherently weaker immune system in men, could contribute to the development of neoplasia.

OTA is transported by the organic anion transporter 1 (OAT1), encoded by the SLC22A6 gene [160]. This transporter is expressed in various tissues, including the liver, small intestine, kidneys, choroid plexus, and blood–brain barrier, highlighting its crucial role in the absorption, distribution, and elimination of xenobiotics [161]. Even low doses of OTA have been shown to upregulate OAT expression [162]. OAT proteins, alongside ABC transporters, play a critical role in the intracellular transport of various substances. Specifically, OAT transporters located in the basolateral membrane of hepatocytes facilitate the uptake of compounds from the bloodstream into liver cells [161]. Numerous studies have demonstrated that OAT expression is androgen-dependent, resulting in significantly higher levels in males. Additionally, it has been observed that men often exhibit higher CYP1A2 enzyme activity compared to women [163]. The study by Ayed-Boussema et al. demonstrated that OTA activates this enzyme, indicating potential differences in OTA metabolism between genders [164]. Interestingly, the study by Pfohl-Leszkowicz et al. confirmed that accelerated metabolism of OTA, associated with increased expression of CYP450 isoforms, predisposed individuals to the development of tumors in the kidneys and urinary tract [165]. This suggests that increased cellular transport of OTA, along with differences in its metabolism, may lead to greater accumulation of OTA in cells and stronger toxic, including carcinogenic, effects. However, establishing the sex-specific causes of cancer incidence requires further intensive research.

## 4. Interactions between Ochratoxin A and Cytostatic Agents

Cytostatic agents, frequently employed in anticancer therapy, may interact with OTA, presenting a significant clinical challenge due to their shared nephrotoxic properties and the potential for mutual potentiation of toxicity, particularly with prolonged exposure [166,167].

Cisplatin is a commonly used chemotherapeutic agent for treating various solid tumors, either alone or in combination with other drugs. Despite its effectiveness, it carries a significant risk of severe side effects, particularly nephrotoxicity. The concurrent use of cisplatin and OTA raises concerns due to the potential for enhanced nephrotoxic effects, which can worsen renal damage [168]. OTA primarily harms the proximal renal tubules, while cisplatin increases nephrotoxicity through ROS production and the induction of apoptosis in renal cells. This simultaneous exposure can lead to compounded renal injury, elevating the therapeutic risks for oncology patients. Additionally, OTA contributes to oxidative stress, resulting in mitochondrial dysfunction and DNA damage in renal cells, further increasing the likelihood of renal failure and irreversible organ damage [168,169,170].

In addition to cisplatin, several cytostatic agents can interact with OTA, producing complex toxicological effects, particularly impacting the kidneys, liver, and cells vulnerable to carcinogenesis. The combination of OTA with doxorubicin, a chemotherapeutic agent that intercalates with DNA and induces ROS production, exacerbates nephrotoxic and hepatotoxic outcomes. This co-exposure amplifies DNA damage and heightens cytotoxicity in healthy cells, thereby increasing the risk of acute kidney injury and compromising overall cellular integrity [171,172]. Moreover, 5-fluorouracil (5-FU) is an antimetabolite that inhibits DNA synthesis, primarily in cancer cells, thereby arresting their proliferation and inducing apoptosis. Although 5-FU is not inherently nephrotoxic, its co-administration with OTA, which induces renal damage, may lead to a synergistic enhancement of toxicity. Studies suggest that OTA can intensify the cytotoxic effects of 5-FU, particularly in renal tissue, where OTA accumulation and oxidative stress are elevated, thereby increasing the potential for renal injury [173,174]. Similarly, methotrexate, a dihydrofolate reductase inhibitor that disrupts folate metabolism and DNA synthesis in rapidly proliferating cells, is known for its nephrotoxic potential, particularly at high doses. This nephrotoxicity is primarily due to the formation of methotrexate precipitates within the renal tubules, leading to cellular damage. Given that OTA also accumulates in the kidneys and causes cellular injury, the co-administration of OTA with methotrexate could exacerbate nephrotoxicity, necessitating close monitoring of renal function to mitigate the risk of acute renal damage [175,176]. Furthermore, cyclosporine A, an immunosuppressant that inhibits T lymphocyte activation, is well-documented for its nephrotoxic potential, particularly with long-term use. The co-administration of OTA with cyclosporine A may further aggravate renal impairment, especially during prolonged exposure, as both agents accumulate in the kidneys and inflict cellular damage, heightening the risk of chronic nephrotoxicity and renal dysfunction [177].

Given these interactions, intensive monitoring of renal function is imperative for patients exposed to OTA while undergoing cytostatic therapy. Furthermore, it is crucial to explore protective strategies to minimize nephrotoxicity. Current research into the management of OTA-induced carcinogenesis focuses on various renal protective approaches, including the use of antioxidants and inhibitors of oxidative stress. These strategies aim to mitigate the risks associated with the toxic interactions between OTA and cytostatic agents, potentially improving patient outcomes and preserving renal health [102].

## 5. Conclusions/Limitations

The results of ochratoxin studies conducted in various animal species may not be fully representative of humans due to significant interspecies differences in metabolism, pharmacokinetics, and sensitivity to the toxin. These differences can lead to variability in the target organs affected by ochratoxin, thereby impacting the applicability of animal research findings to human health. This variability complicates the selection of appropriate laboratory animals, as it is crucial to choose a species that is less predisposed to neoplastic changes unrelated to the substance being tested. Additionally, accurately determining the individual effects of ochratoxin is challenging due to its frequent co-occurrence with other mycotoxins in food. Interactions among these toxins can produce synergistic effects, where the presence of other toxins enhances the impact of ochratoxin. This complicates the assessment of ochratoxin’s effects in isolation and may obscure the true health risks associated with it.

The lack of a definitive conclusion regarding the impact of OTA on carcinogenesis might be attributed not only to the absence of a single specific mechanism responsible for tumor development but also to OTA’s detrimental effects on a wide range of biological processes. Consequently, its carcinogenic potential likely results from the simultaneous influence of multiple factors. Furthermore, studies demonstrating the association between OTA exposure and carcinogenicity are predominantly pathomorphological in nature, while research focusing on cytotoxicity adopts a more molecular approach. This existing disparity hinders the ability to conclusively establish a direct link between the observed disturbances and tumor formation.

## Data Availability

Not applicable.

## Abbreviations

AhR: aryl hydrocarbon receptor; AIDS: acquired immunodeficiency syndrome; AIF: apoptosis-inducing factor; Akt: protein kinase B; Al-NTA: aluminum nitrilotriacetate; BEN: Balkan Endemic Nephropathy; CEG: commonly expressed miRNA groups; COX: cyclooxygenase; CYP450: cytochrome P450; DEG: differentially expressed miRNA groups; EDI: Estimated Daily Intake; EFSA: European Food Safety Authority; EPO: erythropoietin; ERK1-2: extracellular-regulated kinase 1-2; Fe-NTA: iron nitrilotriacetate; FPG: formamido-pyrimidine-DNA-glycosylase; GBC: gallbladder cancer; GCLC: glutamate-cysteine ligase catalytic subunit; GSA: Ghana Standards Authority; GSH: glutathione; HATs: histone acetyltransferases; HCC: hepatocellular carcinoma; HIF1α: hypoxia-inducing factor 1; HO-1: heme oxygenase-1; HPV: human papillomavirus; HSP90: heat shock protein 90; IARC: International Agency for Research on Cancer; IDH1: isocitrate dehydrogenase 1; MAP: Mitogen-Activated Protein; MAPK: Mitogen-Activated Protein Kinase; MDA: malondialdehyde; MOE: Margin of Exposure; NLR: NOD-like receptor; NLRP3: NLR family pyrin domain containing 3; NQO1: NAD(P)H quinone dehydrogenase 1; Nrf1: NF-E2-related factor 2; OAT1: organic anion transporter 1; OGDH: α-ketoglutarate dehydrogenase; OP-OTA: lactone-opened ochratoxin A; OSCC: Ocular squamous cell carcinoma; OSSN: ocular surface squamous neoplasia; OTA: ochratoxin A; OTA-HRP: ochratoxin A conjugated to horseradish peroxidase; PA: penicillic acid; PDK1: pyruvate dehydrogenase kinase 1; PFT: pifithrin-; PHE: L-β-phenylalanine; PI3K: phosphatidylinositide 3-kinase; PTPC: permeability transition pore complex; PXR: pregnane X receptor; RCC: renal cell carcinoma; ROS: reactive oxygen species, SCC: squamous cell carcinoma; TCA: tricarboxylic acid; TCC: Threshold of Toxicological Concern; TDI: Tolerable Daily Intake; TGFβ: transforming growth factor β; TNFα: tumor necrosis factor α; VEGF: vascular endothelial growth factor; VHL: von Hippel-Lindau syndrome; 5-FU: 5-fluorouracil; Δψm: mitochondrial membrane potential.
